# Dichlorofluoromethane: MAK Value Documentation, addendum – Translation of the German version from 2024

**DOI:** 10.34865/mb7543e11_1ad

**Published:** 2026-03-30

**Authors:** Andrea Hartwig, MAK Commission

**Affiliations:** 1 Institute of Applied Biosciences, Department of Food Chemistry and Toxicology, Karlsruhe Institute of Technology (KIT) Adenauerring 20a, Building 50.41 76131 Karlsruhe Germany; 2 Permanent Senate Commission for the Investigation of Health Hazards of Chemical Compounds in the Work Area, Deutsche Forschungsgemeinschaft, Kennedyallee 40, 53175 Bonn, Germany. Further information: Permanent Senate Commission for the Investigation of Health Hazards of Chemical Compounds in the Work Area | DFG

**Keywords:** Dichlorofluoromethane, H-CFC, toxicity, ban of use, Luft, air

## Abstract

The German Senate Commission for the Investigation of Health Hazards of Chemical Compounds in the Work Area (MAK Commission) reviewed its toxicological evalu­ation of dichlorofluoromethane [75-43-4]. Dichlorofluoromethane and other partly halogenated chlorofluorocarbons are no longer approved in the European Union or in Germany. The derivation of the previous MAK value of dichlorofluoromethane does not correspond with the current approach of the Commission. There are no new studies that would allow the MAK value to be revised. The MAK Commission decided that a new evaluation is not of high priority. The MAK value and the other classifications are therefore suspended, and the substance is listed in the Section II c of the List of MAK and BAT Values for substances no longer evaluated.

**Table:** 

**MAK value (2023)**	**See Section II c of the List of MAK and BAT Values**
**Peak limitation**	**–**
	
**Absorption through the skin**	**–**
**Sensitization**	**–**
**Carcinogenicity**	**–**
**Prenatal toxicity**	**–**
**Germ cell mutagenicity**	**–**
	
**BAT value**	**–**

In 1982, a MAK value of 10 ml/m^3^ was derived for dichlorofluoromethane (Henschler 1983, available in German only), and in 2001, Peak Limitation Category II with an excursion factor of 2 was established (Greim 2001, available in German only). In a 90-day inhalation study in rats, gross-pathological changes in the liver were observed at concentrations of 500 ml/m^3^ and above; these did not yet occur at 50 ml/m^3^. The study was not available to the Commission in the original; it was thus not possible to ascertain whether the lowest concentration tested of 50 ml/m^3^ represents the NOAEC (no observed adverse effect concentration). The MAK value was therefore only provisional. The same applies for the peak limitation category.

Dichlorofluoromethane, also known as R21 or Freon 21, belongs to the class of partly halogenated chlorofluorocarbons (H-CFCs). Until 2014 it was used as a cooling agent in refrigeration systems and as an inert propellant. Its use has since been banned due to its ozone-depleting potential (UBA 2014, 2022).

Persistent compounds containing chlorine and bromine such as chlorofluorocarbons and halons can damage the strato­spheric ozone layer. In 1987, the Montreal Protocol was therefore drawn up to protect the ozone layer. This was put in force in Europe and Germany by various regulations (UBA 2022). As a result, the use of this ozone-depleting H-CFC has been banned in Europe since 1 January 2015. The ban on use includes also refilling the systems with used refrigerant and all maintenance and repair work which requires the refrigeration cycle to be interrupted, such as replacing the filter drier or an oil change. This is laid down by Regulation (EC) No 1005/2009 on substances that deplete the ozone layer (European Parliament and European Council 2009). The German federal states are responsible for enforcing the ban (UBA 2014).

The method used to derive the MAK value for dichlorofluoromethane does not correspond to the current approach of the Commission. There are no new studies available that would allow the health hazard to be assessed. Re-evaluation of the substance is at present not of high priority. The MAK value and the peak limitation category have therefore been suspended and the substance has been assigned to Section II c of the List of MAK and BAT Values. This comprises substances for which the MAK values and classifications have been suspended and which at present are not being re-evaluated.
